# Detection of Novel Variations Related to Litter Size in *BMP15* Gene of Luzhong Mutton Sheep (*Ovis aries*)

**DOI:** 10.3390/ani11123528

**Published:** 2021-12-10

**Authors:** Ran Di, Fengyan Wang, Ping Yu, Xiangyu Wang, Xiaoyun He, Joram Mwashigadi Mwacharo, Linxiang Pan, Mingxing Chu

**Affiliations:** 1 Key Laboratory of Animal Genetics, Breeding and Reproduction of Ministry of Agriculture and Rural Affairs, Institute of Animal Science, Chinese Academy of Agricultural Sciences, Beijing 100193, China; diran@caas.cn (R.D.); wangfy504@163.com (F.W.); yuping202106@163.com (P.Y.); wangxiangyu@caas.cn (X.W.); hedayun@sina.cn (X.H.); 2 Small Ruminant Genomics, International Center for Agricultural Research in the Dry Areas (ICARDA), Addis Ababa P.O. Box 5689, Ethiopia; j.mwacharo@cgiar.org; 3 Animal and Veterinary Sciences, SRUC and Centre for Tropical Livestock Genetics and Health (CTLGH), The Roslin Institute Building, Midlothian EH25 9RG, UK; 4 Shandong Yingtai Agriculture and Animal Husbandry Technology Co., Ltd., Jinan 271114, China; yingtai2020@163.com

**Keywords:** sheep, litter size, *BMP15*, *FecB*, *GDF9*

## Abstract

**Simple Summary:**

*BMP15* is a critical gene in sheep reproduction. Most of its variations have been reported in European sheep. In this study, the entire open reading frame (ORF) region of *BMP15* was sequenced in 154 Luzhong mutton sheep. Among 13 identified variations, six were novel. Four SNPs (ENSOART00000010201.1:c.352+342C>A, c.352+1232T>C, c.352+1165A>G and c.353-2036T>A) were significantly associated with litter size, and could be used as candidate genetic markers for improving litter size. The results also suggested possible interaction between *BMP15* and *FecB*/*GDF9*.

**Abstract:**

Litter size is an important economic trait in the mutton sheep industry. *BMP15* is one of the key candidate genes for litter size in sheep. In this study, the entire ORF region of *BMP15* was sequenced in 154 Luzhong mutton ewes, and the novel variations were determined. The association between polymorphism in *BMP15* and litter size was analyzed using a general linear model. Six out of a total of thirteen variations were identified to be novel. Association analysis indicated that four (SNPs ENSOART00000010201.1:c.352+342C>A, c.352+1232T>C, c.352+1165A>G and c.353-2036T>A) were significantly associated with litter size. The joint analysis among three major genes (*BMP15*, *BMPR1B* and *GDF9*) exhibited significant interaction effects in three combinations (*FecB* and c.352+1232T>C of *BMP15*; *FecB* and c.352+1165A>G of *BMP15*; c.352+342C>A of *BMP15* and ENSOART00000014382.1:c.994G>A of *GDF9*). For the SNPs c.352+1232T>C and c.352+342C>A, the global distribution of allele frequencies showed that the highest variation frequency occurs in Western Europe. In conclusion, the results demonstrated that *BMP15* is a major gene for litter size in Luzhong mutton sheep and candidate SNPs associated with litter size were identified.

## 1. Introduction

Litter size has always been the focus of attention for sheep breeders. Increasing litter size is an effective way to improve the economic benefits of the mutton industry. However, the molecular mechanism for litter size has not yet been fully revealed and few molecular markers for litter size have been identified. This hinders the implementation of molecular marker-assisted selection targeting litter size.

*BMP15* is a member of the TGF-β (transforming growth factor β) superfamily [1], and it is a crucial gene in sheep reproduction. The *BMP15* gene is mapped on the sheep X chromosome and comprises two exons and one intron. *BMP15* is mainly expressed in the oocyte, and promotes the proliferation of granulosa cells and the expansion of the cumulus [2,3]. For *BMP15* gene mutations (*FecX^I,H,B,G,L or R^*), heterozygous ewes have a significantly higher number of ovulations or litter size than wild-type ewes; however, homozygous ewes are infertile [4,5,6,7,8]. This may be because heterozygous genotypes of *BMP15* increase the sensitivity of ovarian follicles to follicle-stimulating hormone (FSH) and luteinizing hormone (LH), which can cause antral follicles to develop in advance and discharge multiple oocytes simultaneously [9,10,11]. Therefore, *BMP15* plays a key role in ovulation number and litter size in sheep and is an important candidate gene to identify new molecular markers for litter size. The variations previously found in *BMP15* have mainly been reported in European sheep breeds [5,6,7,8,12,13], but rarely in Chinese sheep.

To date, the BMP signaling pathway has been found to be the most important pathway for the fertility of ewes, and contains most of the major genes for high fecundity (*BMP15*, *GDF9* and *BMPR1B*). In this pathway, BMP15 and GDF9 are ligands, and BMPR1B acts as a receptor. Studies have shown that the combination of these genes has a synergistic effect on sheep litter size. For example, in the Small-Tailed Han, Belclare and Cambridge breeds, the litter sizes of ewes with variations in both *BMP15* and *BMPR1B* or both *BMP15* and *GDF9* were significantly higher than those of ewes with either of the mutations separately [5,14]. In *BMPR1B*, the *FecB* mutation (A746G, Q249R) is the most observed in highly prolific sheep breeds [15,16,17,18]. It is the major mutation responsible for high litter size in Merino sheep [19], Chinese Small-Tailed Han sheep [14,20,21] and Hu sheep [19,22].

The Luzhong mutton sheep is a new breed, and it has high fertility and meat production performance. It was developed by crossbreeding Hu sheep from China with White-headed Dorper sheep from South Africa [23]. Litter size records of Luzhong mutton sheep showed large variations between individual ewes, ranging from one to four. Therefore, the breed is an appropriate candidate for determining the molecular markers encoding the litter size. The patriline of Luzhong mutton sheep is the African Dorper sheep, and previous studies have shown that *BMP15* variations exist in African sheep breeds [24,25]. Therefore, the first objective in the present study was to detect the polymorphism in the open reading frame (ORF) of *BMP15* and assess its association with litter size in Luzhong mutton sheep. Our previous study identified multiple variations in *GDF9* of Luzhong mutton sheep [23]. On the other hand, the maternal Hu sheep carries a high frequency of *FecB* mutations [26,27]. It is therefore reasonable to hypothesize that Luzhong mutton sheep may carry *FecB* and variations of *BMP15* and *GDF9* simultaneously and warrant investigation. The second purpose of this study was to reveal the interaction effects between *BMP15* and *GDF9* genes. Additionally, for individual variations which are associated with litter size, we also mapped the frequency of their distribution across the world.

## 2. Materials and Methods

### 2.1. Animals and DNA Extraction

We sampled 154 3-year-old Luzhong Mutton ewes from the same sheep farm (Shandong Yingtai Agriculture and Animal Husbandry Technology Co. Ltd., Jinan, Shandong, China). This farm adopts the method of natural random mating in the sheep population. Jugular vein blood samples were collected and their litter size at the second parity was recorded. Genomic DNA was extracted from the blood samples using the phenol–chloroform method. All experimental procedures employed in this study were approved by the Animal Welfare Division of the Institute of Animal Science, Chinese Academy of Agricultural Sciences (IAS-CAAS) (Beijing, China). In addition, the ethics approval number is IAS2020-64 (given by the animal ethics committee of IAS-CAAS) on 27 April 2020.

### 2.2. Full-Length Sequencing and Polymorphism Detection of the BMP15 ORF Sequence

To amplify the whole ORF (including all exons and introns) of *BMP15* (GenBank NC_040278.1), ten primer pairs were designed using Primer Premier software (version 5.0, Premier biosoft international Co., Palo Alto, CA, USA). Detailed information of the primers is shown in Table 1, and the locations of individual PCR primers in the *BMP15* gene are shown in Figure S1. Using the Sanger sequencing method, PCR products were sequenced in Beijing Tianyi Huiyuan Biotechnique Co. Ltd. (Beijing, China). There is an overlapping area between every two adjacent PCR products, so the entire *BMP15* ORF sequence was assembled (Table S1). Next, the alignment was performed between *BMP15* gene sequences of the 154 Luzhong ewes and the reference sequence (GenBank Accession number NC_040278.1) using MEGA7 software [28]. Finally, variations of this gene were ascertained in Luzhong mutton sheep and were further retrieved in the Ensembl database (https://asia.ensembl.org/Ovis_aries/Gene/Variation_Gene/Table?db=core;g=ENSOARG00000009372;r=X:50970938-50977454;t=ENSOART00000010201, accessed on 23 February 2021) to ascertain their novelty.

### 2.3. Statistical Analysis

Allele and genotype frequency, heterozygosity (*He*), number of effective alleles (*Ne*) and polymorphism information content (*PIC*) were calculated for *BMP15* using the following formula:

(1) $$He=1-\overset{n}{\underset{i=1}{\sum }}p_{i}{}^{2}$$

(2) $$Ne=1/\overset{n}{\underset{i=1}{\sum }}p_{i}{}^{2}$$

(3) $$PIC=1-\overset{n}{\underset{i=1}{\sum }}p_{i}{}^{2}-\overset{n-1}{\underset{i=1}{\sum }}\overset{n}{\underset{j=i+1}{\sum }}2p_{i}{}^{2}p_{j}{}^{2}$$

where *n* is the number of alleles, *p_i_* is the allele frequency of the *i*th allele and *p_j_* is the allele frequency of the *j*th allele.

The test for deviation from Hardy–Weinberg equilibrium was performed for genotype distribution of each locus with the chi-square test [29]. The association of *BMP15* genotypes with litter size was analyzed using the general linear model in R software (aov, Version 4.0.3). The least-squares means of litter size were used for multiple comparisons among the different genotypes with Tukey HSD test. The fixed model was *y* = *μ* + *G1* + *G2* + *G1G2* + *e*, where *y* is the phenotypic value of litter size, *μ* is the population mean, *G1* is the fixed effect for *FecB* genotype or *GDF9* genotype, *G2* is the fixed effect for *BMP15* genotype, *G1G2* is the fixed interaction effect of *FecB* and *BMB15* combined genotypes or *GDF9* and *BMB15* combined genotypes and *e* is the residual error. The *GDF9* and *FecB* data were analyzed in our previous report [23], and results indicated that the SNPs ENSOART00000014382.1:c.994G>A (rs421019907) and c.978A>G (rs399579080) in the *GDF9* gene were significantly associated with litter size. Because these two SNPs are linked, we selected one of them (c.994G>A) to analyze its interaction with the SNPs in *BMP15*.

For SNPs which are significantly associated with litter size and have been annotated in the Ensembl database, we downloaded their allele frequencies in breeds from the International Sheep Genome Consortium and the NextGen Project, and genotyped them in 9 Chinese breeds (*n* = 40–100 in each breed) and Australian Merino sheep (*n* = 50) using Sanger sequencing and the same primers used in Luzhong sheep. Combining all the above data, we constructed the global geographic distribution of the allele frequencies for these SNPs.

## 3. Results

### 3.1. Detection of BMP15 Polymorphism in Luzhong Sheep

**Figure 1:**
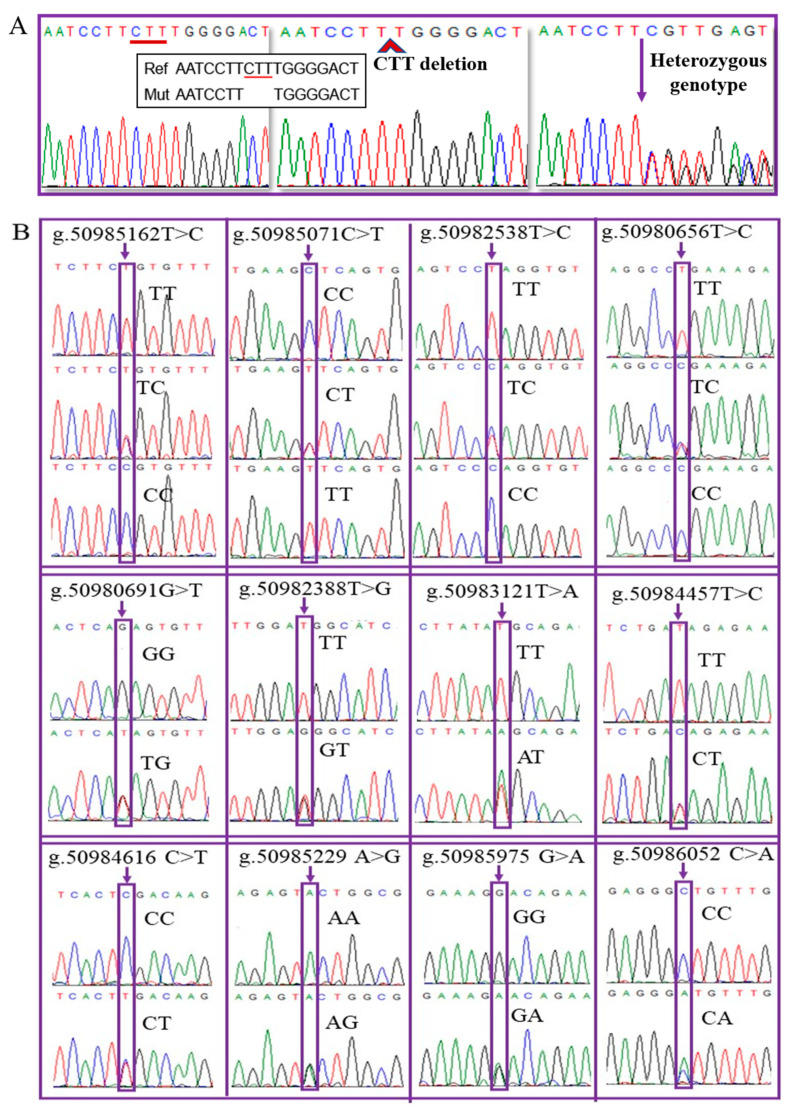
Sequence profiles of 13 variations in the *BMP15* gene in Luzhong mutton sheep. (**A**) Sequence profile of the CTT base deletion at the ENSOART00000010201.1: c.58_60 in exon 1. (**B**) Sequence profile of the 12 base substitutions in intron 1 and exon 2.

The entire ORF region of *BMP15* was sequenced in 154 ewes with different litter sizes. By comparing these sequences with the *BMP15* reference sequence of Texel sheep (GenBank: NC_019484.2), 13 variations were observed. One CTT base deletion (ENSOART00000010201.1: c.58_60del) was detected in exon 1, and its sequence profile is shown in Figure 1A. A total of 12 base substitution variations were identified in intron 1 (10 SNPs) and exon 2 (2 SNPs), and their sequence profiles are shown in Figure 1B. Among these variations, six are novel and have not been reported in the Ensembl database (Table 2). Variation c.782T>C altered the amino acid (Leu (L)–Pro (P)) at residue 252, and c.747G>T variation altered the amino acid at residue 240 (Gln (Q)–His (H)).

### 3.2. Population Genetic Analysis of SNPs in BMP15 Gene in Luzhong Sheep

Results of population genetic analysis showed that four SNPs (c.352+1232T>C, c.352+1323C>T, c.353-1453T>C and c.782T>C) were moderately polymorphic (0.25 < *PIC* < 0.5), and eight other SNPs (c.352+342C>A, c.352+419G>A, c.352+1165A>G, c.352+1778C>T, c.352+1937T>C, c.353-2036T>A, c.353-1303T>G and c.747G>T) had low polymorphisms (*PIC* < 0.25) in Luzhong mutton sheep (Table 3). Additionally, the chi-square test indicated that eleven SNPs were in Hardy–Weinberg equilibrium (*p* > 0.05), except c.782T>C and c.58_60del (*p* < 0.05).

### 3.3. Association of Polymorphisms in BMP15 with Litter Size in Luzhong Sheep

The association analysis indicated that the four SNPs (c.352+342C>A, c.352+1232T>C, c.352+1165A>G and c.353-2036T>A) in *BMP15* were significantly associated with litter size in Luzhong ewes (Table 4). For SNPs c.352+342C>A and c.353-2036T>A, the litter sizes of ewes with the heterozygous genotype were the highest and were significantly higher than those of ewes with the homozygous genotype (*p* < 0.05). There were no combination effects among the different SNPs of *BMP15* on litter size. In addition, the interactions between *FecB* or c.994G>A of the *GDF9* gene and four variations associated with litter size in *BMP15* were assessed, and the results showed significant interaction effects (*p* < 0.05) in three combinations (*FecB* and c.352+1232T>C, *FecB* and c.352+1165A>G, c.352+342C>A of *BMP15* and c.994G>A of *GDF9*; Table 5).

### 3.4. Geographic Distribution of Allele Frequency of Two Variations in the BMP15 Gene

**Figure 2:**
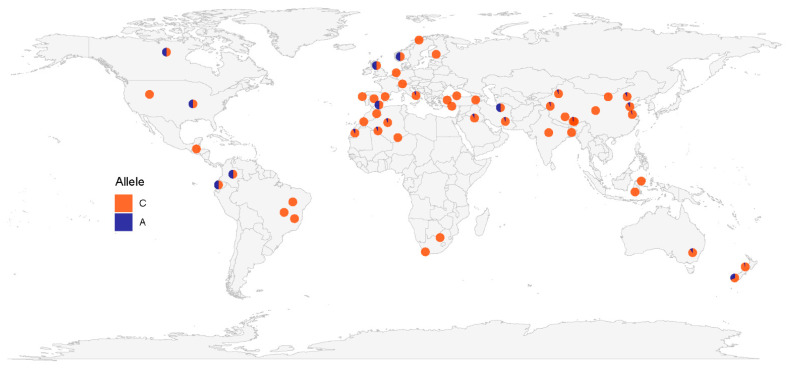
Allele frequency distribution of the variation c.352+342C>A in the *BMP15* gene.

**Figure 3:**
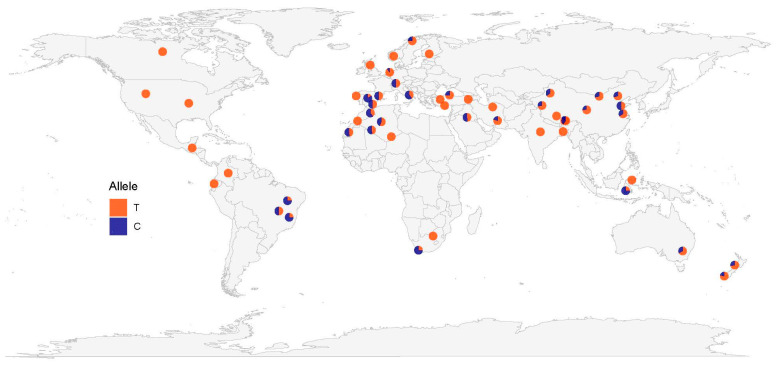
Allele frequency distribution of the variation c.352+1232T>C in the *BMP15* gene.

Of the SNPs that were significantly associated with litter size, two variations (c.352+342C>A and c.352+1232T>C) have been annotated in the Ensembl database. Therefore, the allele frequencies of these two SNPs in breeds from the International Sheep Genome Consortium and the NextGen Project databases can be downloaded and used. Combining this data with our sequence data for nine Chinese sheep breeds and Australian Merino sheep, the global geographic distribution map of the allele frequencies for these two SNPs was constructed. The results (Figure 2) showed that the highest frequency (f = 0.5) of the c.352+342C>A variation occurs in sheep of Western Europe, America, and Iran. The frequency of this variation in New Zealand is also relatively high (f = 0.311), however its frequency in Asia and Africa is low (f < 0.1). The variation c.352+1232T>C occurs more widely and its frequency is comparatively high across all continents (Figure 3). Its frequencies are the highest in southwestern Europe (f = 0.5–0.833), Indonesia (f = 0.75), South Africa (f = 0.75), northwestern Africa (f = 0.52–0.6) and eastern South America (f = 0.5–0.75). Its frequency in Asia and Australia is also relatively high.

## 4. Discussion

As an important member of the BMP/SMAD signaling pathway, *BMP15* plays a significant role in influencing litter size and ovulation rate in sheep. In the present study, we identified 13 variations (including 6 new ones) in the entire ORF region of the *BMP15* gene, and 4 were significantly related to litter size. Of these 13 mutations, 7 are consistent with those reported in the variant table of the sheep *BMP15* gene in the Ensembl database (http://asia.ensembl.org/Ovis_aries/Gene/Variation_Gene/Table?db=core;g=ENSOARG00000009372;r=X:50970938-50977454;t=ENSOART00000010201;v=rs592773279;vdb=variation;vf=27853699 (accessed on 20 November 2021)). Among them, two variations (ENSOART00000010201.1: c.58_60del and c.782T>C) can cause amino acid changes. The first deletion variation is widespread globally with a relatively high frequency (http://asia.ensembl.org/Ovis_aries/Variation/Population?db=core;g=ENSOARG00000009372;r=X:50970938-50977454;t=ENSOART00000010201;v=rs592773279;vdb=variation;vf=27853699 (accessed on 20 November 2021)). Demars et al. [12] and Hanrahan et al. [5] reported no significant associations between multiple births and this mutation in Cambridge, Belclare, Olkuska and Grivette sheep. The frequency of this variation in the Finnish Landrace, Finnish Landrace × Texel-cross and the composite sheep was 0.23, 0.08 and 0.32, respectively. Among them, the variation was only associated with litter size in the composite sheep (*p* < 0.001) [30]. In Xinjiang Cele Black Sheep, a significant difference in litter size was reported between the deletion homozygous genotype and the heterozygous genotype; however, no significant differences existed between other genotypes [31]. Therefore, the impact of this variation on litter size is complex, and more breeds will need to be analyzed to estimate its effects. The second variation (c.782T>C) has a high frequency (>0.5) in Bangladeshi, Garut, Ronderib Afrikaner and Sumatran sheep (http://asia.ensembl.org/Ovis_aries/Variation/Population?db=core;g=ENSOARG00000009372;r=X:50970938-50977454;t=ENSOART00000010201;v=rs55628000;vdb=variation;vf=27853624 (accessed on 20 November 2021)). It has not been reported in relation to litter size or the number of ovulations in sheep. The other six variations were detected for the first time in this study and are thus lacking in the Ensembl database.

For ENSOART00000010201.1:c.58_60del and c.782T>C, with no correlation to litter size as observed in this study, they were in Hardy–Weinberg disequilibrium, suggesting that their frequency may be affected by artificial selection or genetic drift. These two variations can result in amino acid changes that likely affect protein function; therefore, they may be key variation sites responsible for other traits and could be the targets of artificial or natural selection. Although there is no correlation between them and litter size, they warrant further investigation to ascertain their possible association with other traits.

In this study, the four SNPs that were significantly related to litter size are located in the intron of *BMP15*. However, most of the previously reported *BMP15* variations related to litter size are in the exon. They included: *FecX^I^* (detected in New Zealand sheep), *FecX^H^* (New Zealand) [4], *FecX^B^* (Ireland), *FecX^G^* (Ireland and Iran) [5], *FecX^L^* (France) [6], *FecX^R^* (Spain) [7,9,12], *FecX^R^*^A^ (Spain) [32], *FecX^Bar^* (Tunisia) [13] and AF236078.1:c.379G>A(Glu41Lys) (Iran) [33]. In addition, mutations in the regulatory region of this gene can also affect litter size. For instance, in the Noire du Velay (NV) breed, an SNP (*FecX^N^*, OARX:50977717T>A) upstream of *BMP15* was found to be extremely significantly correlated with litter size through genome-wide association analysis, and the mutation could reduce the *BMP15* promoter activity, which affects the expression of the gene in oocytes leading to an increase in litter size [34]. Using selection signature analysis, Dolebo et al. identified one significant candidate region on the X chromosome overlapping *BMP15* in the African multi-lamb Bonga breed, implying this gene could be a key candidate fertility gene in the breed [25]. For variations in introns, it is relatively difficult to analyze their function. These polymorphisms may affect miRNA regulation, enhancer function, degree of glycosylation etc., which require further experimentation to verify.

In this study, the geographic distribution of allele frequencies for SNPs c.352+342C>A and c.352+1232T>C in *BMP15* indicated that Western Europe has the highest variation frequency for these two SNPs, although the detailed worldwide distributions of the allele frequencies are distinct for the two variations. This result coincides with reports on polymorphisms in this gene showing that its variations mainly occur in European sheep [4,5,9,10,11,12,13,28,29,32,34,35,36], which implies that *BMP15* is a key gene for high litter size of European sheep.

BMP15, GDF9 and BMPR1B play a role in the BMP/SMAD signaling pathway, simultaneously. BMP15 and GDF9 are secreted by oocytes, and they act through binding to receptors (e.g., BMPR1B) located on the granulosa cells, then signals are further transmitted via phosphorylation of SMAD2/3 [37]. They work together to regulate cell differentiation, follicular atresia and oocyte maturation [38].Therefore, *BMP15*, *GDF9* and *BMPR1B* may have synergistic effects on sheep ovulation rate [11]. Previous studies showed that the combined effect of *BMP15* and *FecB* mutation appears to have a multiplier effect, indicating a functional interaction between *BMP15* and *BMPR1B* [39]. We previously found that ewes with *FecB* and BMP15 (*FecX^G^*) mutants had a higher litter size than those carrying individual gene mutants in Small-Tailed Han sheep [14]. In addition, ewes carrying both *FecB* and *BMP15* (*FecX^I^*) variations have extremely high ovulation rates (258% of controls) in 1/4 Merino 3/4 Romney sheep [40]. In this study, *BMP15* and *FecB* also show synergistic effects on litter size, but the locations of the *BMP15* variation (c.352+1232T>C and c.352+1165A>G) differ from those of previous reports. Studies have also shown that BMP15 and GDF9 are closely related and are very likely to interact. GDF9 and BMP15 can heterodimerize to form an effective growth factor accumulating protein. In the accumulation complex, BMP15 “activates” the potential GDF9 to produce effective signaling in granulosa cells via activin receptor-like kinase-4/5 (ALK4/5) and SMAD2/3 transcription factors, promoting the expansion of mouse cumulus cells and improving the quality of oocytes in vitro [41]. Peng et al. also found that the GDF9-BMP15 heterodimer was a more biologically active ligand compared to the homodimer in mice and humans [37]. Therefore, the interaction between *GDF9* and *BMP15* may impact litter size and ovulation rate in sheep. In Cambridge and Belclare sheep, ewes carrying both *GDF9* and *BMP15* mutations have higher ovulation rates than ewes carrying individual gene mutants [5]. In this study, our results also indicated a significant interaction effect between c.352+342C>A of the *BMP15* gene and c.994G>A variation of *GDF9*. Together, these results confirm that GDF9 and BMP15 play a synergistic role as ligands in the BMP/SMAD pathway. The pathway will be severely inhibited if *GDF9* and *BMP15* are mutated simultaneously, which ultimately leads to an increase in ovulation [12].

In the current study, the second parity data were collected and analyzed. However, it is essential to analyze the data of multiple parities for obtaining more accurate results in future studies.

## 5. Conclusions

In this study, a total of 13 variations, including 6 novel variations, were identified in the entire ORF region of the *BMP15* gene. Among the variations, four (c.352+342C>A, c.352+1232T>C, c.352+1165A>G and c.353-2036T>A) were significantly associated with litter size in Luzhong mutton sheep. These results imply that *BMP15* is a critical gene for the litter size of Luzhong mutton sheep, and these four SNPs can be used as candidate variants for improving litter size. The results suggest an interaction effect between *BMP15* and *FecB*/*GDF9* in influencing litter size.

## Figures and Tables

**Table 1:** Amplification primers for the *BMP15* gene in Luzhong mutton sheep.

Primer Name	Primer Sequence (5′–3′)	Annealing Temperature/°C	Amplified Fragment/bp
*BMP15*-F1	CTGCCTGCCAGCCTTTCAT	60	718
*BMP15*-R1	ACATCAATGAGTTGCCCTG		
*BMP15*-F2	GGGAAAACCGCACCATTG	60	843
*BMP15*-R2	CAGAAGCAACTATGAGGGAA		
*BMP15*-F3	GGCTGTTTGTCTTGTTTTAT	60	892
*BMP15*-R3	GAAGATGTGGGTTTGAT		
*BMP15*-F4	AGGTGTGTGTGCGAACTCAG	60	488
*BMP15*-R4	CATTTGCTGTGCTGTACCAC		
*BMP15*-F5	CATCTTCCGTGTTTCCT	60	703
*BMP15*-R5	CCTATTTCATTCTTTGGT		
*BMP15*-F6	AGGCATTGTTCTAGGTGTTGG	60	1084
*BMP15*-R6	CCTTTTACCTGCTGGTAAACAC		
*BMP15*-F7	TTCCTGGCCCTGATCCTTAG	60	1021
*BMP15*-R7	CACTGTTTCCCCATCTATTTGC		
*BMP15*-F8	GTTCATGGATTCAGTGGAGAAGG	60	1179
*BMP15*-R8	CCAAACTGTGATGCTGACACC		
*BMP15*-F9	GTTTGGGTGAACTCCAGGAGT	60	1061
*BMP15*-R9	CATTTTGCAGACGAGGAAACT		
*BMP15*-F10	TGTATTTGAGGTGTTTTTCTCCG	60	1381
*BMP15*-R10	AAGTACAATGCTGAAGGCAAGG		

**Table 2:** The detailed information of 13 variations in the *BMP15* gene in Luzhong sheep.

Region	Location in ENSOART000 00010201.1	Genomic Location (ChrX: Oar_v4.0)	Wild	Mutant	Amino Acid Change	Variations in Ensembl Database
Exon 1	c.58_60del	50986688–50986686	CTT	del	Leu (L)11 del	Yes (rs592773279)
Exon 2	c.782T>C	50980656	T	C	Leu (L) 252 Pro (P)	Yes (rs55628000)
	c.747G>T	50980691	G	T	Gln (Q) 240 His (H)	No
Intron 1	c.353-1303T>G	50982388	T	G	-	No
	c.353-1453T>C	50982538	T	C	-	Yes (rs403715147)
	c.353-2036T>A	50983121	T	A	-	No
	c.352+1937T>C	50984457	T	C	-	No
	c.352+1778C>T	50984616	C	T	-	No
	c.352+1323C>T	50985071	C	T	-	Yes (rs420350765)
	c.352+1232T>C	50985162	T	C	-	Yes (rs400940002)
	c.352+1165A>G	50985229	A	G	-	No
	c.352+419G>A	50985975	G	A	-	Yes (rs412881200)
	c.352+342C>A	50986052	C	A	-	Yes (rs426251007)

**Table 3:** Population genetic analysis for variations of *BMP15* in Luzhong mutton sheep.

Variations	Genotype Frequency			Allele Frequency		*PIC*	*He*	*Ne*	Chi-Square Test (*p*-Value)
c.352+342C>A	CC	AC	AA	C	A				
	0.844(130)	0.156(24)	0.000(0)	0.922	0.078	0.133	0.144	1.168	0.294
c.352+419G>A	GG	AG	AA	G	A				
	0.974(150)	0.026(4)	0.000(0)	0.987	0.013	0.025	0.026	1.026	0.870
c.352+1165A>G	AA	AG	GG	A	G				
	0.935(144)	0.065(10)	0.000(0)	0.967	0.033	0.061	0.063	1.067	0.677
c.352+1232T>C	CC	CT	TT	C	T				
	0.234(36)	0.532(82)	0.234(36)	0.500	0.500	0.375	0.500	2.000	0.420
c.352+1323C>T	CC	CT	TT	C	T				
	0.299(46)	0.558(86)	0.143(22)	0.578	0.422	0.369	0.488	1.952	0.073
c.352+1778C>T	CC	CT	TT	C	T				
	0.987(152)	0.013(2)	0.000(0)	0.993	0.007	0.013	0.013	1.013	0.935
c.352+1937T>C	TT	CT	CC	T	C				
	0.974(150)	0.026(4)	0.000(0)	0.987	0.013	0.025	0.026	1.026	0.870
c.353-2036T>A	TT	AT	AA	T	A				
	0.961(148)	0.039(6)	0.000(0)	0.980	0.020	0.038	0.038	1.040	0.805
c.353-1453T>C	CC	CT	TT	C	T				0.073
	0.234(36)	0.571(88)	0.195(30)	0.520	0.480	0.375	0.499	1.997	
c.353-1303T>G	TT	GT	GG	T	G				0.935
	0.987(152)	0.013(2)	0.000(0)	0.994	0.007	0.013	0.013	1.013	
c.747G>T	GG	GT	TT	G	T				0.497
	0.896(132)	0.104(22)	0.000(0)	0.948	0.052	0.094	0.099	1.109	
c.782T>C	CC	CT	TT	C	T				0.000
	0.013(2)	0.662(102)	0.325(50)	0.344	0.656	0.349	0.451	1.823	
c.58_60del	CTT/CTT	CTT/---	---/---	CTT	---				0.001
	0.234(36)	0.623(96)	0.143(22)	0.545	0.455	0.373	0.496	1.984	

**Table 4:** Least-square means and standard errors of litter size for different genotypes in Luzhong ewes.

Variation	Genotype	Litter Size (Mean ± SD)
c.352+342C>A	CA(24)	2.083 ^a^ ± 0.647
	CC(130)	1.531 ^b^ ± 0.648
c.352+419G>A	GG(150)	1.620 ^a^ ± 0.681
	AG(4)	1.500 ^a^ ± 0.535
c.782T>C	CT(102)	1.686 ^a^ ± 0.702
	CC(2)	1.500 ^a^ ± 0.000
	TT(50)	1.460 ^a^ ± 0.610
c.353-1453T>C	CC(36)	1.611 ^a^ ± 0.595
	TT(88)	1.833 ^a^ ± 0.785
	CT(30)	1.545 ^a^ ± 0.657
c.352+1323C>T	CC(46)	1.652 ^a^ ± 0.636
	TT(22)	1.558 ^a^ ± 0.623
	CT(86)	1.773 ^a^ ± 0.912
c.352+1232T>C	TT(36)	1.861 ^a^ ± 0.827
	CT (82)	1.639 ^ab^ ± 0.635
	CC (36)	1.500 ^b^ ± 0.591
c.352+1165A>G	AA(144)	1.645 ^a^ ± 0.683
	AG(10)	1.200 ^b^ ± 0.410
c.352+1778C>T	CC(152)	1.612 ^a^ ± 0.670
	CT(2)	2.000 ^a^ ± 1.155
c.352+1937T>C	TT(150)	1.613 ^a^ ± 0.672
	CT(4)	1.750 ^a^ ± 0.886
c.353-2036T>A	TT(148)	1.608 ^b^ ± 0.675
	TA(6)	1.833 ^a^ ± 0.718
c.353-1303T>G	TT(152)	1.625 ^a^ ± 0.678
	TG(2)	1.000 ^a^ ± 0.000
c.747G>T	GG(132)	1.674 ^a^ ± 0.684
	GT(22)	1.125 ^a^ ± 0.336
c.58_60del	CTT/---(96)	1.573 ^a^ ± 0.643
	---/---(22)	1.727 ^a^ ± 0.634
	CTT/CTT(36)	1.667 ^a^ ± 0.787

Note: Different letters (a, b) for litter size indicates significant differences (*p* < 0.05). The number after the genotype denotes the number of ewes with a different genotype.

**Table 5:** Results of interactive effects between *FecB* or *GDF9* c.994G>A and four variations associated with litter size in the *BMP15* gene.

*FecB* or *GDF9* c.994G>A Variation	*BMP15* Variation	*p*-Value
*FecB*	c.352+342C>A	0.147
*GDF9* (c.994G>A)		0.039
*FecB*	c.352+1232T>C	0.041
*GDF9* (c.994G>A)		0.805
*FecB*	c.352+1165A>G	0.010
*GDF9* (c.994G>A)		0.448
*FecB*	c.353-2036T>A	0.772
*GDF9* (c.994G>A)		0.170

## Data Availability

Not applicable.

## Supplementary Materials

The following are available online at https://www.mdpi.com/article/10.3390/ani11123528/s1, Figure S1: locations of individual PCR primers in the BMP15 gene, Table S1: Sequence of the entire ORF region of the BMP15 gene in Luzhong mutton sheep.
