# Supplementary material for: Detection of Novel Variations Related to Litter Size in BMP15 Gene of Luzhong Mutton Sheep (Ovis aries)
Source: Animals (Basel). 2021 Dec 10;11(12):3528. doi: 10.3390/ani11123528 (PMC8698048; doi:10.3390/ani11123528)
Supplement: Supplementary file 1 [file animals-11-03528-s001.zip › Table S1.pdf]

animals

MDPI

Supplementary Materials

Detection of Novel Variations Related to Litter Size in *BMP15* Gene of Luzhong Mutton Sheep (*Ovis aries*)

Ran Di [1,†], Fengyan Wang [1,†], Ping Yu [1], Xiangyu Wang [1], Xiaoyun He [1], Joram Mwashigadi Mwacharo [2,3], Linxiang Pan [4] and Mingxing Chu [1,*]

1 Key Laboratory of Animal Genetics, Breeding and Reproduction of Ministry of Agriculture and Rural Affairs, Institute of Animal Science, Chinese Academy of Agricultural Sciences, Beijing 100193, China; diran@caas.cn (R.D.); wangfy504@163.com (F.W.); yuping202106@163.com (P.Y.); wangxiangyu@caas.cn (X.W.); hedayun@sina.cn (X.H.)
2 Small Ruminant Genomics, International Center for Agricultural Research in the Dry Areas (ICARDA), P.O. Box 5689 Addis Ababa, Ethiopia; j.mwacharo@cgiar.org
3 Animal and Veterinary Sciences, SRUC and Centre for Tropical Livestock Genetics and Health (CTLGH), The Roslin Institute Building, EH25 9RG, Midlothian, Scotland
4 Shandong Yingtai Agriculture and Animal Husbandry Technology Co., Ltd., 271114 Jinan, China; yingtai2020@163.com
* Correspondence: mxchu@263.net; Tel.: +86-010-6281-9850
† These authors contributed equally to this work.

Citation: Di, R.; Wang, F.; Yu, P.; Wang, X.; He, X.; Mwacharo, J.M.; Pan, L.; Chu, M. Detection of Novel Variations Related to Litter Size in *BMP15* Gene of Luzhong Mutton Sheep (*Ovis aries*). *Animals* **2021**, *11*, 3528. https://doi.org/10.3390/ani11123528

Academic Editors: Maria Consuelo Mura, Sebastiano Luridiana and Giovanni Cosso

Received: 26 October 2021
Accepted: 5 December 2021
Published: 10 December 2021

Publisher's Note: MDPI stays neutral with regard to jurisdictional claims in published maps and institutional affiliations.

Copyright: © 2021 by the authors. Submitted for possible open access publication under the terms and conditions of the Creative Commons Attribution (CC BY) license (https://creativecommons.org/licenses/by/4.0/).

Table S1. Sequence of the entire ORF region of the BMP15 gene in Luzhong mutton sheep.

Gene	Sequence of the entire ORF region of the BMP15 gene in Luzhong mutton sheep
BMP15(Ovis aries)	ATGGTCCTCCTGAGCATCCTTAGAATCCTTTGGG-<br>GACTGGTGCTTTTTATGGAACATAGGGTCCAAATGACACAGGTAGGGCAGCCCTC-<br>TATTGCCCACCTGCCTGAGGCCCCTACCTTGCCCCTGATTCAGGAGCTGCTAGAAGAA-<br>GCCCCTGGCAAGCAGCAGAGGAAGCCGCGGGTCTTAGGGCATCCCTTACGGTATATGCTG-<br>GAGCTGTACCAGCGTTCAGCTGACGCAAGTGGACACCCTAGGGAAAACCGCACCATT-<br>GGGGCCACCATGGTGAGGCTGGTGAGGCCGCTGGCTAGTGTAG-<br>CAAGGCCTCTCAGAGGTGAGTTATCATACTATATTGTTCTGGTGGGAGGGGGGA-<br>GAAAATGGGGAAGAAAAGTGTAGAAAAAAGTGGATCTGTCAG-<br>TTTTCTGTCAGGCTTCACATTGCCTACAGGGTAGGTGGTTTTCAAAAGATGGCACCCTTGGGA-<br>GAACCTGGCTCCAAATTTGCTTCCCTTTAGGGCTCCAATTTAAGAACAGATTGCCTT-<br>GGGGCCTCCCTGAGGACTTTCTCCCAGGCCCCAGGGCAACTCATT-<br>GATGTGTCAGCTCCTAGCTTTCAAAGAAAGTTTTAATGCTTATTATATCAC-<br>TGAAAATTATAACTGAGAAATACTCATTATGTTCTGTGGAGGGCTGTTTGTCTT-<br>GTTTTATTCTTAGAAGACAAAAGGTACAGGATGTTGAGTTGAG-<br>GAAATTTACAAGGCTTCAGAAAGGACAGAAACAGGCTCACACCTGAGAG-<br>TCTGTTATTGTATTTAATCCTTAATGA-<br>TAGGGACTCTCCTTAGCTGTCCTGAAAGGCTTAGCCTGGTTTTTAAAGAAAAGGGAAACAGA-<br>GAGCAAAGTTAAATGACTTGCAGAGGTCACAAGGATGGGGTGGCAGAGGCAGGAGTT-<br>GAGCTTCAAGTCTGTCTGATTTTATAGTCTGTCCATAACTACTTAAGTGTTTTGAATGTCAT-<br>ACCCTGATATCCTTGTACACGAAAGCAGTTTTTCTGTTTAGACTATGTTGCTAGTTT-<br>GGGTTTGTGGTACCAAATGTATTTTGGAATTTTCTTTGTTTCCCTCATAGTT-<br>GCTTCTGCTTTAAACATCTTCATTAGAGACTGATCTAGGGCAATAGTGGGCTTATGCAGTG-<br>CAGGGTTCTCTAGGGAATGAAGAAAGCCATCAATTTCAAGGAAATCTTGGGAAC-<br>CTGGTGAAATCAATCTTAAGCCTTTGCTAGTCTCAAAGATGACACCAGACATGATTTAC-<br>CTCCCTCCTCCAAGTAAAACAACCACCATTCCCTTAAGCTAAACTTTAGTGTGTGCTTAC-<br>CATGTGCCGGGTATTAGGCACTGTACTTTTACAAACATTATCTTCCTTAATCCTCCCCAA-<br>TATCATATGAAGTAGGTGTGTGTGCGAACTCAGTTGTATCTGATTCTTTGTGACCCCATGGAC-<br>TATACCTGCCAGGCTCCTCTGTCCATGGAACTTTCCAGGCAAGAGTACTGGCGTGGGTT-<br>GCCATTTCCTCCTCCAGGG-<br>GATCTTCCCATCCCAGGGATCAAACCCACATCTTCTGTGTTTCCTGCATTGGCAAGAGGAT-<br>TCTTTACTACTGCTCCACTATGGAATACTATGAATATACCCATTATATAGGTGAA-<br>GAAAATGAAGCTCAGTGTAACACAGCTAGTAATTAATAGAGCTGGAT-<br>TTCTAACCCGGGCTTTCAGGCTTCAGAGCCTGTGCTTTTAATTACCCTGCTGAGCTGACTGCT-<br>TAACAGACTTTAATTGAGCACCAAGCACTGTGTTGGGGTTGGGTATAAAAAGGCAAA-<br>TAACTGCTCGAGGAGCCCCCACTATAGTAGAATGGATATATAAGTAACAACATAATGGTGG-<br>TACAGCACAGCAAATGCTTTGAAAAGGATAAACAGTAGGATTCCTGGGGCTCCAAGAA-<br>GAAATGGGTTGCTACTTAGGGCAAGGAGAGGGAAATGCTGAAATAGTTGAAAGGCCTCATT-<br>GGAGAGGCAGCATTTGAGCCAGACTTTGCAGAAGGAGTAAAGAGACTATGGATA-<br>CAGGGAGGGAAGTGCATTCTGATTGAGGAAAAAGAAGATAACTTGATAAGGTTGGAGAT-<br>TCACTCGACAAGTATTTATTTAGCAACTGTTAAGTGCTAGGCATTGTTCTAGGTGTT-<br>GGAATGTGATCAAGATGGGCACGGCTCTCTGGGTGAGACAAGTAAGTAAACATGGATAGTAG-<br>TAAATTGTGGTAAATGCTACCAAAGAATGAAATAGGTGGTTCTGATAGAGAATAGCAAA-<br>GAAATCTTCCCTTCGCATGGGATTGTCAGTGAAGGCATGTTTGAAGAGGTGACATTT-<br>GAGCTGAGGTCTGTAAGGTAAGAAGCTTGGCATGAGAAGAGAGGAGA-<br>GAATTCTGATCTAGGCAGAGGGAACAACATGTACAAAGGCCTAAAGGTGGAAACAAGTACTT-<br>GAAGAATAGAAGGAAGATCAGGATGGTTGGAGCCTAGTGAGATTGTAGAGAGTCGTGGGA-<br>TATGAAACTGATGAGGCTCAGGGATGCAGTGGTCATTACAAGCGATCTGGATTTCAC-<br>TCATAAGCTGCATAAGTCAATTCTGTACAGATGATATGAGGCTAGAAAGTAGAATGTCTA-<br>GAATTAGGCCAGTATCTTGATCTGACCATGGGACATACTGG-<br>TATCTAGGGACTCTGAAAAGGATCCAGAAATGGATGACAAGAGAGTT-<br>GGGTCTGATCATTCCATCAGAGAGATGATCCAGCTGAATCAAGCTTGCTTTGAGAGC-<br>TATTCCAGGTTCAATCGCACAGGATGTGTGAGTCAGACATTTCCCTGAAAAGTTCTGAG-<br>CAGACCCTTCCTGGCCCTGATCCTTAGCAACCCCACCCTGCTTCAGGAAA-

TATGTGATGTCAGGGTCTGAAAGGAAAGGGCAGTTCCTCACCAGACTGCCTGCTAGAGAT-
TCGGTTTAGATTCCTTTTCAGGCAGGGTGGGGCAGATGTATGGGTGGGTGTCTTGGAAAGAT-
TATTACAGGATGGGAGTATGGAGTAACTCGAGAAAGCAATTACAAGCCTGGCTTGAGCTTT-
GTCTGAATGATATCCAAGTACCCCAAACTTGAGGGTGTATTAAGCATGAGTTGGAACCTGAAC-
TAAAAAAAAAAAATTGATGTATGGTTTCACTCATGTGGAA-
TATAAAAACAAAAATTAAAAACTCAAACAGATCAGATTAGTGTTTACCAG-
CAGGTAAAAGGGTTGGAGAGTGTGCGAAATAGGTGAAGGGAGTTAATTGTGTGG-
TAACAGATGATGGTAACTAAGCTTGTGGTGGTGATCACTCTGTAGTGTATACAGATGTTGAAT-
TATGAATTATGATTATGAATTTTGCACCTGAAACTTGGATA-
CATAACTAAAAAAATCCTGATGCATCAAAACATTCAAGAGCTTCTATGTTGAACATCCCAA-
GCTTACTGAATTCCAGGTTCATGGATTCAGTGGAGAAGGAAAATTAGGTAGATAA-
TATGTAAATTAGCATGGCCATGGTTAGTGACTGGACATGGAAAAACAGACTGGTTCTAAA-
TAGGAAAAGGAGTACGTCAAGGCTGTATATCGTCACCCTGCTTATTTAACTTATATGCAGAG-
TACATCATGAGAAACACTGGGCTGGAAGAAACACAAGCTGGAATCAAGATTGCCGGGACAAA-
TATCAATAACCTGAGATATGCAGATGATACCACCCTTATGGCAGAAAGTGAAGGGGAACTAAA-
GAGCCTCTTGATGAAAGTGAAAGAGGAGAGTGAAAAAGTTGGCTTAAA-
GCTCAACATTCAGAAAACTAAGATCATGGCATCAGGTCCCATCACTTCATGGCAAA-
TAGATGGGGAAACAGTGACAGACTTTATTTTTCTGGGCTCCAAAATCACTG-
CAGATGGTGACTGCAGCCATGAAATTAAAATAAATAAGACACTTGCTCCTT-
GGAAGGAAAGTTATGACCAACCTAGATTGCATATTAAAAAGCAAAGACATTATTTT-
GCCAACAAAGATCCATCTAGTCAAGGCTATGGTTTTTCCAGTAGTCATGTATGGATGTGA-
TAGTTGGACTATAAAGGAAGCTGAGCGCTGAAGAATTGATGCTTTTGGACTGTGGTATTGGA-
GAAGACTCTTGAGAGTCCCTTGGACTGCAAGGTGATCCAACCAGTCCATCCTGGGG-
GAAATCAGTCCCAGGTGTTCATTGGAGGGACTGATGTTGAAGCTGAAACTCCAATACTTT-
GGCCACCTGATGTGAAGAGCTGACTAATTGGAGATGACCCTGATGCTGTGAAAGATTGGGGG-
CAGGAGGAGAAGGGGACAACAGAGGTTGAGATGGTTGGATGGCATCAC-
CAACTCAATGGACATGGGTTTGGGTGAACTCCAGGAGTT-
GGTGATGGACAGGGAGGCCTGGCGTGTTTTGGTTCATGGGGTCACAAAGAGTCGAACAC-
GACTGAGCAACTGAACTGAACTGATGGTTAGTGATAGGTGAA-
GATGACAGAAGGGTTTTTCTTGCTATTGACCTTTTTTGTAATCGTTGCTCTTGACCTTT-
GCCAGGAGAGGGCGCAGAAGGTCAGAGCACTGCAGGTCCTGCTTTTTAACGCTTTGGCCAC-
TAGGTGTCAGCATCACAGTTTGGATTAAAATAATCACTTTGTAGCTCAGCTGGTAAA-
GAATCCGCCTGCAATGCGAGAGACCTCAGTTCGATCCCTGGGTTGGGAAGATCGCTGGA-
GAAGGGAAAGGCTACCCACTCCAGTATTCTGGCCTGGAGAATTCTATGGACTG-
TATAGTCCATGAGGTCGCAAAGAGTCAGACAAGGCTGAGCGACTTTCACTTTCAAAAGTT-
GCATCTTTTAAGTAGACTTTTTAGTTGAGGTCATTTATATGACTGGGCCTTGTTGAAAACGG-
TATTTACAATGAATGGGGTAAATGAAGAATCATTTCCAGTAAGGAATGGAAAAGAGGGA-
GATTGAGGATTAAGGGAGAACCTGCCCAATAATGGAATAGCTGCTCAAGAAGAAATTCAAA-
TAGAAAGCAAAGTATTAAATACCTGTCCCAACTAGGAAAAATTATTTTGTGG-
CATCTCCAACCCAGATTTGAAGCAATAGCAGACCATACCCCCCAA-
TATTGGACTGTCACCCTAAAGCTGCCACTATATAGTGATATAGCATGAGGATTTATTAATTCG-
TATGTTTCAATGATCCTCTTAATTGGTCACCTTTTTAATAGTCAGCTAAA-
TAATACAATATATACAGACAGTTCTGTATTTGAGGTGTTTTTCTCCGTCTAGGGGTATGAG-
TGATCTAAAAATGAGCCACAATTTGTCATCTTAAGGGAAAAAGACTT-
GGACTCAAATCTTTATTCTAACAAACACTGGCTTGTGTGTCCTCTGG-
CATAGCTTCTCTGAGCTTCAGTTTCCTCGTCTGCAAAATGGGAATAGCAAC-
TATCTCATAAGGCTATTGTGGATTCAAGAGCAAATGCATGTAAAGCATCTAATACATTATA-
TAAGTGCTCAATAGATCGCTATTATGATCTTAAATTCATCTCAAGGCTGCTTGTCAGTTT-
GTACTGAGCAGGTCTGTTAGAGAGACTAAGGCTAGGATATAAGAAGCTAACGCTTTGCTCTT-
GTTCCCTCTTACTAATGCAGGCTCCTGGCACATACAGACCCTGGACTTTCCTCTGAGAC-
CAAACCGGGTAGCATACCAACTAGTCAGAGCCACTGTGGTTTACCGCCATCAGCTTCAC-
CTAACTCATTCCCACCTCTCCTGCCATGTGGAGCCCTGGGTCCAGAAAAGCCCAAC-
CAATCACTTTCCTTCTTCAGGAAGAGGCTCCTCAAAGCCTTCCCTGTTGCCCAAAACTT-
GGACAGAGATGGATATCATGGAACATGTTGGGCAAAAGCTCTGGAATCACAAGGGGCG-
CAGGGTTCTACGACTCCGCTTCGTGTGTCAGCAGCCAAGAGGTAGTGAGGTTCTTGAG-
TTCTGGTGGCATGGCACTTCATCATTGGACACTGTCTTCTTGTTACTGTATTTCAATGACAC-
TCAGAGTGTTCAGAAGACCAAACCTCTCCCTAAAGGCCTGAAAGAGTTTACAGAAAAA-

	GACCCTTCTCTTCTCTTGAGGAGGGCTCGTCAAGCAGGCAGTATTGCATCG-GAAGTTCCTGGCCCCTCCAGGGAGCATGATGGGCCTGAAAGTAACCAG-TGTTCCCTCCACCCTTTTCAAGTCAGCTTCCAGCAGCTGGGCTGGGATCACTGGATCATT-GCTCCCCATCTCTATACCCCAAACTACTGTAAGGGAGTATGTCCTCGGGTACTACAC-TATGGTCTCAATTCTCCCAATCATGCCATCATCCAGAACCTTGTCAGTGAGCTGGTG-GATCAGAATGTCCCTCAGCCTTCCTGTGTCCCTTATAAGTATGTTCCCATTAG-CATCCTTCTGATTGAGGCAAATGGGAGTATCTTGTACAAGGAGTATGAGGG-TATGATTGCCCAGTCCTGCACATGCAGGTGACGGCAAAGGTGCAGCTAGCTCAGGTTT-GCCCAAGAAATTCGAAAAGGATTTATAATAAATACTGTTAAATCTGAGAG-TGCTCAACCCAAGTGCTCTACCCAATCTGTAGATTCTATTCCTTGCCTTCAGCATT-GTACTTTAAGTCTTCTTCCCCTATTTATGAGTGCCTCACTTTATAAACAGTTCTGATGCCAAA
