# Supplementary figures and images for: Detection of Novel Variations Related to Litter Size in BMP15 Gene of Luzhong Mutton Sheep (Ovis aries)
Source: Animals (Basel). 2021 Dec 10;11(12):3528. doi: 10.3390/ani11123528 (PMC8698048; doi:10.3390/ani11123528)

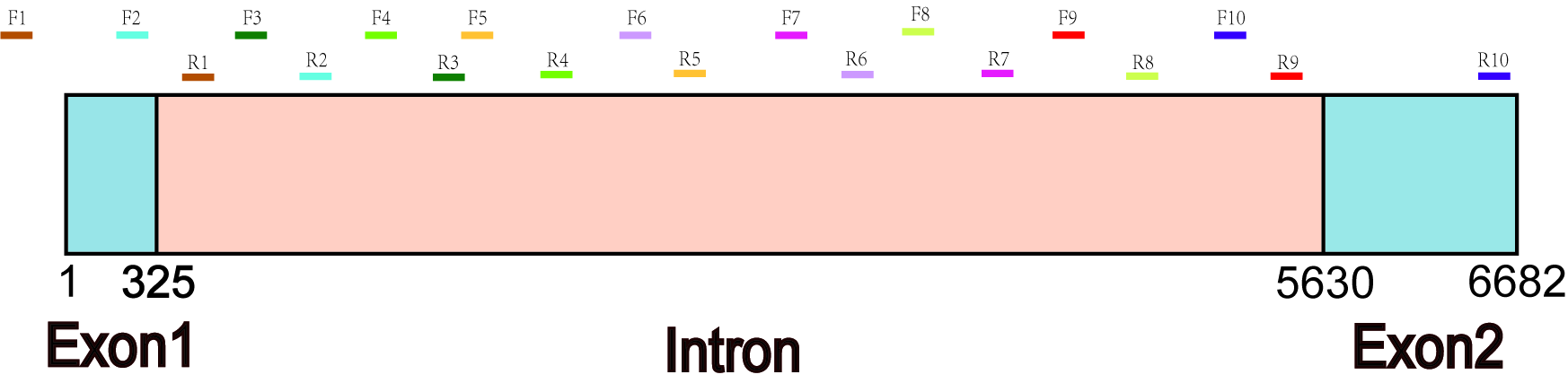

Supplement: Supplementary file 1 [file animals-11-03528-s001.zip › Fugure S1-locations of individual PCR primers in the BMP15 gene.tif]
